# Long-Term Results of Single-Anastomosis Duodeno-ileal Bypass with Sleeve Gastrectomy (SADI-S)

**DOI:** 10.1007/s11695-021-05879-9

**Published:** 2022-01-15

**Authors:** Andrés Sánchez-Pernaute, Miguel Ángel Rubio Herrera, Natalia Pérez Ferré, Carlos Sáez Rodríguez, Clara Marcuello, Clara Pañella, Leyre Lopez Antoñanzas, Antonio Torres, Elia Pérez-Aguirre

**Affiliations:** 1 Department of Surgery, Hospital Clínico San Carlos, C/Martín Lago S/N 28040, Madrid, Spain; 2 Department of Endocrinology, Hospital Clínico San Carlos, Madrid, Spain

**Keywords:** SADI-S, Duodenal switch, Hypoabsorption, Duodeno-ileostomy, Sleeve

## Abstract

**Background:**

Single-anastomosis duodeno-ileal bypass with sleeve gastrectomy (SADI-S) is a simplification of the duodenal switch (DS) in which the alimentary limb is eliminated, and the common channel is lengthened from 200 to 300 cm. Short-term results have demonstrated that SADI-S is safe and reproducible and that weight loss and comorbidities resolution are comparable to biliopancreatic diversion or DS.

**Objective:**

To analyze the long-term outcomes of SADI-S.

**Methods:**

From May 2007 to December 2015, 164 patients were consecutively submitted to a one-step SADI-S. The mean age was 47 years, and the mean body mass index (BMI) was 45.8 kg/m^2^. A total of 101 patients had type 2 diabetes, 91 arterial hypertension, 81 obstructive apnea, and 118 dyslipidemia. Limb length was 200 cm in 50 cases, 250 cm in 99, and 300 cm in 15.

**Results:**

There was no mortality. One patient had a gastric leak, and 2 patients had an anastomotic leak. A total of 25% of the patients were lost to follow-up at 10 years. Excess weight loss and total weight loss were 87% and 38% at 5 years and 80% and 34% at 10 years. A total of 12 patients were submitted to revisional surgery for hypoproteinemia. Preoperatively 41 diabetics were under insulin treatment; at 5 years, 7 remained with insulin and 12 at 10 years. Mean glycemia was 104 mg/dL at 5 years and 118 mg/dL at 10 years. Mean HbA1c was 5.51% at 5 years and 5.86 at 10 years.

**Conclusion:**

In the long term, SADI-S offers satisfactory weight loss and comorbidities resolution.

**Graphical Abstract:**

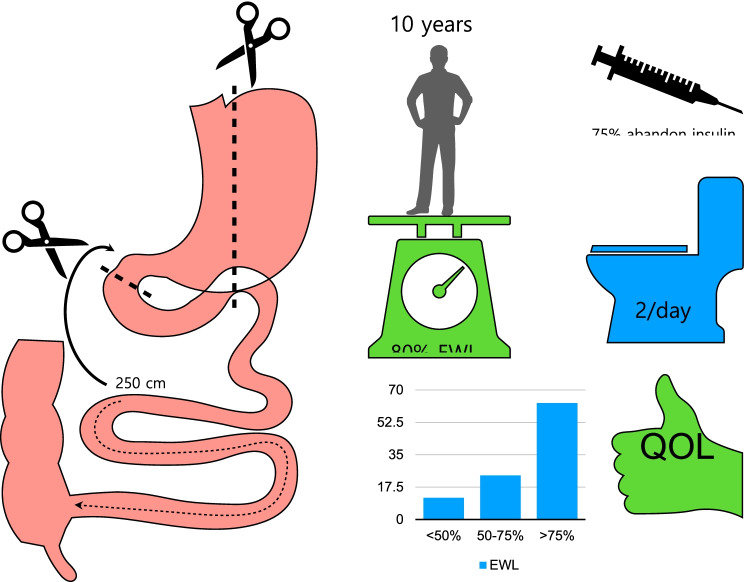

## Introduction

**Fig. 1:**
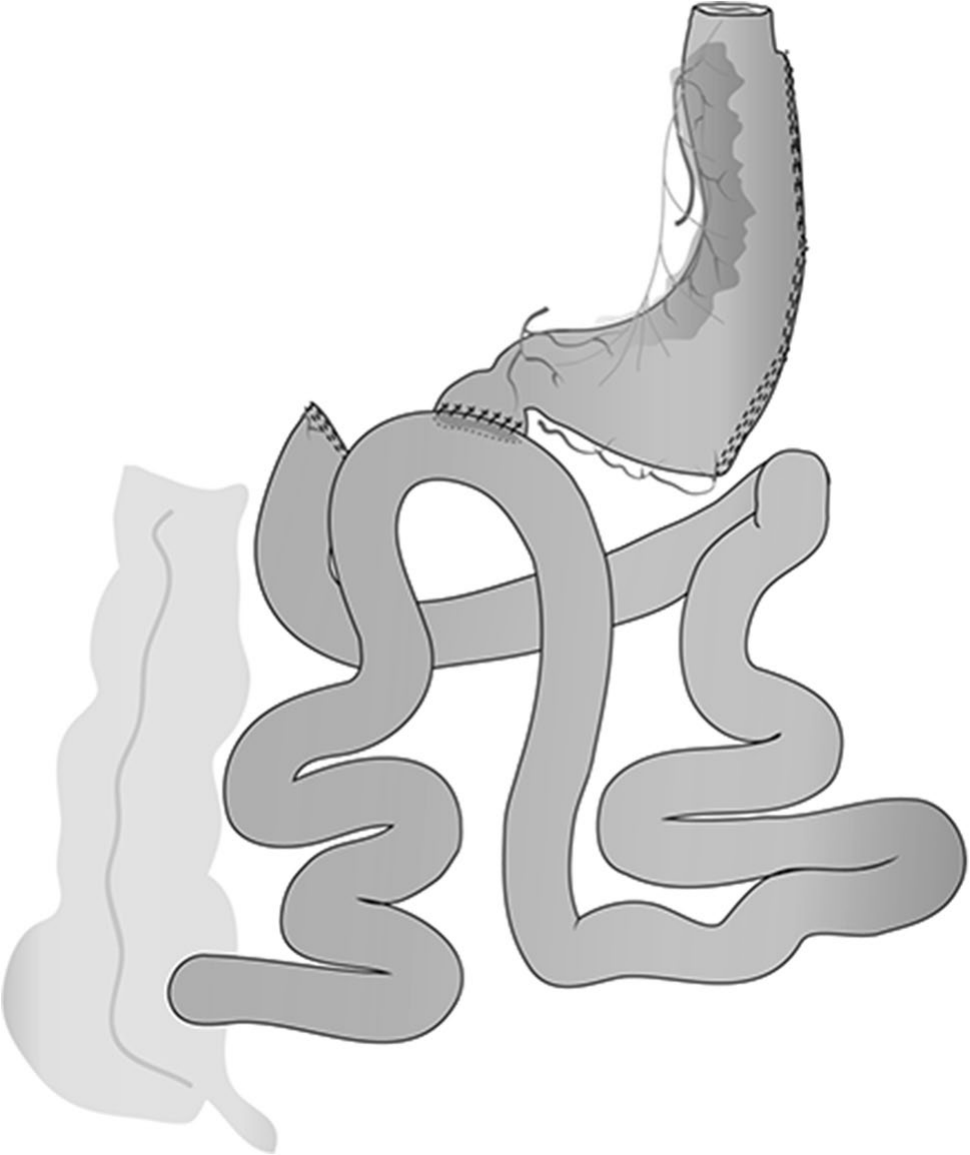
Scheme of SADI-S

The SADI-S (Fig. 1) was introduced in 2007 in our institution [1] with the intention of simplifying a difficult but very effective surgical technique to treat morbid obesity, the DS [2–5]. The operation differed substantially from the previous one-anastomosis technique, the one-anastomosis gastric bypass (OAGB) [6], first because of the pylorus preservation, which warranted the absence of pathologic bile reflux, and second because the common channel length was always measured, as it was in the Scopinaro procedure [7, 8] and the duodenal switch [2], the operations known as biliopancreatic diversions.

SADI-S has been demonstrated to be a safe and reproducible technique [9] and offers good weight loss results in the short term [10, 11]. It results equally effective as a second-step or revisional operation after sleeve gastrectomy [12–14]. Results on diabetes are comparable to those obtained with the duodenal switch [15], and some studies have even found a better metabolic effect thanks to the longer common channel [16].

The present manuscript reviews the experience with the SADI-S in a series of patients operated consecutively in our department and followed for at least 5 years. Second-step and revisional surgeries have been excluded to avoid bias in the interpretation of the results.

## Methods

From May 2007 to December 2015, 199 patients underwent SADI-S, among which 164 had a primary SADI-S and constitute our study group. Indications for single-step SADI-S in our Department are BMI over 45 kg/m^2^ or severe metabolic disease independently of the BMI. The mean age of the patients was 47 years (22–71), the mean weight was 124 kg (72–180) and the mean BMI was 45.8 kg/m^2^ (34–67). There were 99 women and 65 men. Type 2 diabetes was present in 101 cases (61.5%), of which 47 were on oral therapy (46.5%), 42 were on insulin therapy (41.5%), and 12 were under diet control. Arterial hypertension (AT) was present in 91 cases (55.4%), obstructive sleep apnea (OSA) in 81 (49.3), and dyslipidemia in 118 (71.9%).

All patients underwent a thorough endocrine and psychiatric evaluation previous to surgery, as recommended by international guidelines.

The operation has been described elsewhere [1, 10–13] (Fig. 1).

### Postoperative Workup

Patients initiated oral liquid intake and were encouraged to stand and start walking at 6 h from surgery. The drain was removed after intestinal passage was confirmed, usually the 2nd postoperative day, and patients were discharged on a high protein liquid hypocaloric diet. After 2–3 weeks, a pureed/soft food was initiated, and a solid diet with a high protein intake (> 80 g/day) was introduced by the second postoperative month. Visits were scheduled with the nutritionist, the endocrinologist, and the surgeon, at least 4 times in the first and second postoperative year, every 6 months until the 5th year and once yearly thereafter. Laboratory analysis was performed twice per year. Supplementation included initially iron, calcium, vitamin D, and a multivitamin complex. Depending on the laboratory tests, supplements were discontinued or increased.

### Statistics

Continuous variables are presented as mean and standard deviation or median and interquartile range as required. Comparisons were made using the *t*-test or chi-square. SPSS v27 for Mac was used for all calculations. Weight loss is expressed as final BMI, excess weight loss (EWL), and total weight loss (TWL).

### Ethics

All patients gave informed consent to have their data included in a prospective database. The study was registered in ClinicalTrials.gov (NCT01463904). The Institutional Review Board of the Hospital approved the revision of the clinical and surgical reports of all patients. Specific formal consent for this type of study is not required.

## Results

### Surgery and Postoperative Outcome

Primary SADI-S was completed in all 164 patients. The common limb length was 200 cm in 50 cases, 250 cm in 99, and 300 cm in 15. The anastomosis was hand-sewn in 58 cases and stapled (30 mm EndoGIA, Medtronic) in 106. Cholecystectomy was performed in 18 patients. Methylene-blue water-tightness test was systematically performed, and a retroanastomotic vacuum drain was left in all cases. A liver biopsy was routinely performed.

There was 1 gastric leak and 2 duodeno-ileostomy leaks; one of them needed reoperation while the other was managed conservatively. One patient was reoperated for an intestinal perforation, and 2 were submitted to revisional laparoscopy for peritoneal bleeding. One patient presented intraluminal bleeding from the gastric staple line and was submitted to endoscopic treatment. Two patients suffered from abdominal hernia incarceration, one from a preexisting epigastric hernia and the other one from a trocar site hernia; both were reoperated. Two patients underwent long respiratory support, and one needed tracheostomy for weaning. The mean postoperative stay was 7 days. After discharge, 23 patients were attended in the emergency ward in the first month (14%), and 5 were re-admitted (3%) for fever or abdominal pain. Only in one case, an abdominal collection was found, and antibiotic therapy was started.

The operative liver biopsy revealed normal histology in 5.4%, mild steatosis in 48%, moderate in 28%, and severe steatosis or steatohepatitis in 17% of the cases.

### Follow-up and Long-Term Outcome

**Fig. 2:**
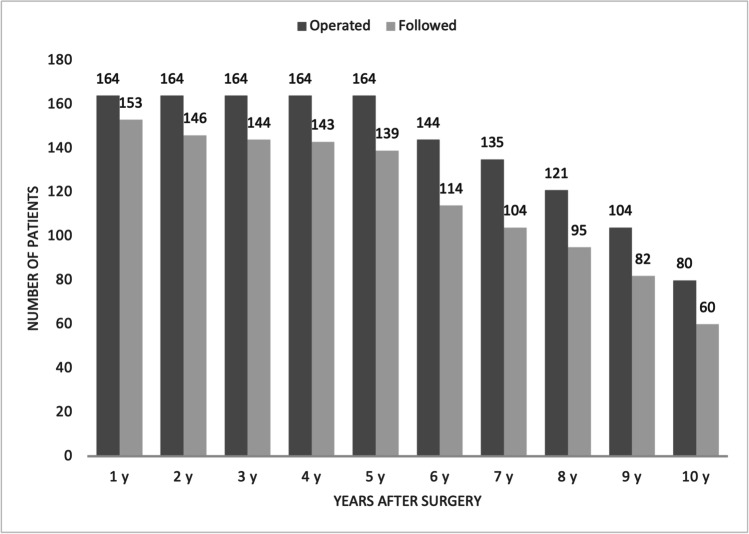
Long-term follow-up

**Fig. 3:**
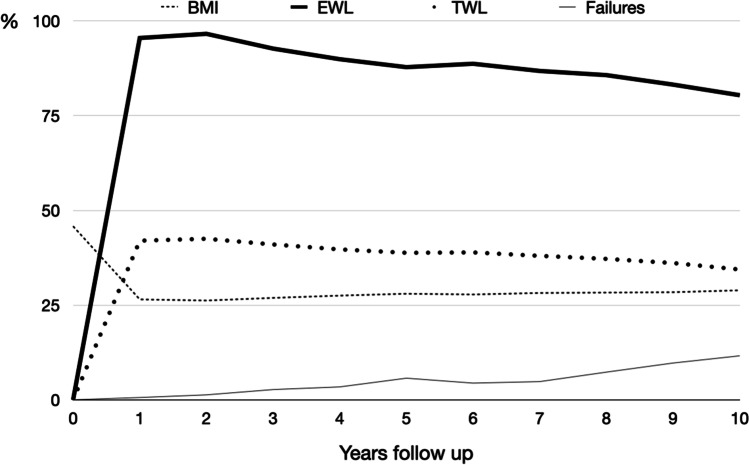
Long-term weight loss. EWL, excess weight loss; TWL, total weight loss; BMI, body mass index. All data are expressed in % except for BMI, which is expressed in kg/m^2^

There was no mortality. Follow-up was 84.7% at 5 years and 75% (60/80) at 10 years (Fig. 2). Weight loss is presented in Table 1 and Fig. 3. Eighty-two percent of the patients maintained vitamin D supplementation in the long term, 75% had calcium, 45% iron, 25% vitamin E, and 20.5% vitamin A. Despite supplementation, deficiencies were frequently seen, particularly in ferritin (66.7%), vitamin D (57.9%), and vitamin A (26,7%). Laboratory tests data are presented in Table 2.

**Table 1:** Weight loss

Time	BMI	EWL (%)	TWL (%)	% failures
Basal	45.8	0	0	0
1 year	26.5	95.5	42	1/153–0.6
2 years	26,2	96.6	42.5	2/146–1.3
3 years	26.9	92.7	41	4/144–2.7
4 years	27.5	89.9	39.7	5/143–3.4
5 years	28	87.8	38.8	8/139–5.7
6 years	27.8	88.7	38.9	5/114–4.4
7 years	28.2	86.8	38	5/104–4.8
8 years	28.3	85.7	37.2	7/95–7.3
9 years	28.4	83.2	36.1	8/82–9.7
10 years	28.9	80.4	34.4	7/60–11.6

*BMI*, body mass index; *EWL*, excess weight loss; *TWL*, total weight loss

**Table 2:** Laboratory tests and deficiencies at 5 and 10 years

		5 years			10 years	
	Mean	Range	% abnormal	Mean	Range	% abnormal
Total protein (g/dL)	6.78	5.7–7.9	25.2	6.8	5.9–8.3	16.3
Albumin (g/dL)	4.09	2.7–5	2.7	4.09	3.6–4.8	0
Hemoglobin (g/dL)	13.08	8.3–16.7	15.9	12.91	9.2–15.7	23.4
Iron (µg/dL)	73.6	12–244	18	67.02	11–130	14.6
Ferritin (ng/mL)	81	2.5–1555	56	47.6	2.5–546	66.7
Folic acid (ng/mL)	7.1	1.5–24.5	13.1	7.3	2.1–20.6	15.9
Vitamin B12 (pg/mL)	398	161–1386	1	455	122–1500	6.8
Calcium (mg/dL)	8.9	6.4–9.9	0	9.02	8.1–9.08	0
Vitamin D (ng/mL)	24.8	3–155	74	32.27	3.4–126	57.9
Parathormone (pg/mL)	112	32–673	77	117	41.2–433	70
Vitamin A (mg/L)	0.34	0.07–0.87	40.8	0.42	0.11–0.94	26.7
Vitamin E (mg/L)	8.1	3–13	8.5	7.43	2.7–12.5	20
Copper (µg/dL)	97.5	17–163	22.7	104.8	63–148	8.7
Zinc (µg/dL)	65.5	47–94	31.8	71.2	50–93	9.1
Selenium (µg/dL)	69	28–108	26	79.5	39–117	17.6

Twelve patients have been submitted to revisional surgery for recurrent hypoproteinemia (7.3%), 7 of them in the initial group with a 200 cm common limb (14%), 5 among those with a 250 cm limb (5%), and none of those with a 3 m common channel. One patient was submitted to re-sleeve for insufficient weight loss.

There were 101 patients with diabetes mellitus, with a mean time from diagnosis of 9 years. The evolution of the disease, as well as arterial hypertension and obstructive apnea, are presented in Table 3. Dyslipidemia improved significantly initially, from a preoperative rate of 72 to 35% at 5 years, although at 10 years, the rate increased to 53% (Table 4).

**Table 3:** Evolution of type 2 diabetes, hypertension, and obstructive apnea

	Preoperative	5 years	10 years
Insulin (*n*)	41	7	12
Oral (*n*)	47	17	27
Diet/no. therapy (*n*)	13	77	62
Glycemia (mg/dL)	169.8 (88–408)	104.16	118.2 (74–207)
HbA1c (%)	7.69 (5.4–14)	5.51	5.86 (4.6–7.9)
Arterial hypertension (%)	56	25.7	14
Obstructive apnea (%)	54	5.8	2.1

**Table 4:** Lipidic profile

		Preoperative			5 years			10 years	
	Mean	Range	% abnormal	Mean	Range	% abnormal	Mean	Range	% abnormal
Triglycerides (mg/dL)	183	50–799	57	90.7	37–232	7	113	49–362	20
HDL (mg/dL)	47.8	23–82	24	53.4	28–85	10.6	54.4	31–92	13
LDL (mg/dL)	105.2	35–197	64	84.4	26–187	23	90.6	21–172	36
Cholesterol (mg/dL)	190	110–313	41	157.2	84–273	8	166.4	100–264	8.5
Dyslipidemia (%)			72			35			53.8

Four patients had a previous cholecystectomy in 18, it was performed along with the weight-loss surgery, because of symptomatic cholelithiasis, and in 11 cases (7.7%) cholecystectomy was performed in the follow-up, only one for acute cholecystitis.

Eleven patients have died in the follow-up for different causes: 4 due to respiratory disease, 1 for end-stage renal disease, 1 after a cerebrovascular accident, 1 for unknown cause peritonitis, and 3 due to neoplastic disease (gastric, bladder, and lung). Four other patients are alive with neoplastic disease, colon, lung, bladder, and melanoma, for a total rate of 4.2% of new tumors diagnosed after the operation and a total incidence density rate of 0.6 cancer cases per 100 person-years. One patient died due to respiratory infection for COVID-19; another one survived the infection after 3 months under ventilatory support. Nephrolithiasis with hyperoxaluria has been detected in 7 cases (4.2%), slightly more frequent between patients with a shorter common limb (6.3% in patients with a 2 m limb).

Only one patient was submitted to reoperation for pathologic gastroesophageal reflux disease (GERD) along with a hiatal hernia; reduction of the hernia and a Hill’s procedure with hiatoplasty was performed. An upper gastrointestinal endoscopy was performed in 36 cases at a mean time of 6 years from the SADI-S. Most of the patients (72%) had a normal endoscopy. Grade A esophagitis was present in 5 cases (14%), and grade C or D in 3 patients (8.3%).

The mean number of stool frequency was 2.4 per day (0–8) at 5 years from surgery and 2.1 (0–6) at 10 years. A small number of patients with steatorrhea underwent dietary counseling plus loperamide or cholestyramine.

## Discussion

The present work reports the 5- to 10-year outcome of a series of patients consecutively submitted to primary SADI-S in one institution, and it constitutes the largest series with more than 5 years of follow-up, 139 patients, and the only one reporting data at 10 years from surgery. SADI-S is a simplification of the DS. Although the procedure has been demonstrated to behave differently to previous malabsorptive procedures—firstly because when the common channel is equal to or longer than 250 cm, it does not give rise to malabsorption—the relation with BPD-DS is so tight that we still consider SADI-S a modification of the former instead of a totally new procedure.

The first challenge of this operation was to demonstrate that weight loss was comparable to that obtained with the DS. The initial common limb was 200 cm, which signified a twofold to fourfold increment from the lengths chosen by Hess [2], but as the alimentary limb had been eliminated, it was difficult to estimate the weight loss after both modifications. The average excess weight loss at two years was 100%. However, some patients developed severe problems derived from malnutrition and had to be revised. In 2009 the common limb length was changed to 250 cm, which is the current standard length, and, in selected cases, to 300 cm. As a whole, patients submitted to SADI-S achieved a 5-year EWL of 87% and a 10-year EWL of 80%. Hess’ report of DS at 10 years [17] with an excellent 92% follow-up rate (167 patients) showed an average EWL of 75%, with a revisional rate of 3.7%, mostly due to undernutrition and protein insufficiency. Comparable weight losses are reported by Marceau and Topart [18, 19]. The only article communicating a significant higher EWL is the work of Bolckmans and Himpens [20], in which they obtain a 93% mean EWL at 10 years performing a DS with a sleeve gastrectomy made over a 34 French bougie, an alimentary limb of 250 cm and a common limb of 75 to 100 cm; the main drawback of their work is that more than 10% of their patients had to be submitted to revisional surgery for undernutrition. Recently Surve et al. have communicated an 80% EWL and 36% TWL at 6 years in a series of 750 consecutively operated patients, among which 46 were followed for more than 6 years [21]. Patients with an initial BMI over 50 kg/m^2^ achieved a significantly lower EWL, 81 versus 90% at 5 years and 75 versus 81% at 10 years; however, when TWL was considered, no differences could be found [22].

Hypoabsorptive surgery offers a greater weight loss than less aggressive techniques, but it has the drawback of inducing a higher rate of nutritional deficiencies and the possible development of malnutrition. To counterbalance this, three rules must be strictly respected: follow-up, dietary compliance, and supplementation. We have attained a 75% follow-up rate at 10 years, which is difficult to improve. Attending to diet and supplementation, patients are encouraged to include in their daily intake at least 100 g of proteins and no more than 30% of fat. Even though supplementation is prescribed to every patient, deficiencies are detected. At 5 years, hypoproteinemia affects 25% of the patients, low vitamin D is detected in 70%, low ferritin in 56%, iron in 18%, vitamin A in 40%, and zinc in 32%. At 10 years, secondary to intestinal adaptation, these numbers are fairly improved. Deficiencies detected are comparable to those reported by Himpens and Topart [19, 20] after the duodenal switch, by Scopinaro after the classical form of biliopancreatic diversion [23] or after the distal gastric bypass [24]. Excluding the initial series with a short common channel, the rate of revisional surgery for undernutrition was 4.3%.

The results of SADI-S on diabetics were expected as biliopancreatic diversions are the most powerful metabolic operations [25, 26]. This is the reason why we offer SADI-S to our diabetic patients independently of the BMI, and so the rate of diabetic patients in the present series is higher than the observed in other works. The anastomosis of the duodenum with the proximal ileum reduces carbohydrate absorption because most of the hexose transporters of the intestinal cells are located in the jejunal mucosa [27]. This is shared by the Scopinaro procedure, DS, and SADI-S, but in this last one, as the common channel is longer glucose absorption is higher what results in a higher postprandial glycemia [16]. On the other hand, the longer absorptive intestinal length increases the time of interaction between the bile acids and the membrane of the enterocytes where the TGR5 receptors stimulate the secretion of GLP-1, hence improving glycemic control in diabetics [16]. Regarding dyslipidemia, although 53% of the patients had some alteration of the lipid profile, the individual analysis revealed triglyceride alterations in 20% of the patients, HDL in 10%, LDL in 36%, and total cholesterol in 8.5%, numbers slightly worse than those reported after DS [20], what is surely secondary to the increased length of the common channel were fat is absorbed.

Intestinal diseases, as inflammatory bowel diseases, mesenteric ischemia, or malabsorptive bariatric operations enhance colonic oxalate absorption in response to fat malabsorption [23, 28], probably secondarily to the binding of colonic fats to free calcium what increases the amount of unbound oxalate that is able to pass through the intestinal mucosa into the bloodstream [29]. After SADI-S, we observed a small number of cases with nephrolithiasis and hyperoxaluria, with a higher rate in patients with a shorter common limb. However, the rate of urolithiasis that we found in our series is not higher than that reported after Roux-en-Y gastric bypass [30].

A frequent concern after sleeve-based operations is the development of gastroesophageal reflux disease. We found advanced esophagitis in only 8.3% of the cases submitted to upper gastrointestinal endoscopy, which is a significantly lower proportion than could be expected, and we attribute it to the wider sleeve gastrectomy associated with the procedure.

SADI-S offers a greater and more stable weight loss in the long term than gastric bypass [31–33]. But more important is the absence of secondary complications requiring reoperation. Obeid NR et al. reported a 20% rate of reoperation in the first 10 to 13 years after a Roux-en-Y gastric bypass for long-term complications of the initial operation, internal hernia, obstruction-, or gastro-gastric fistula [32]. Duvoisin et al. report 14% of complications requiring surgery after gastric bypass, mainly anastomotic complications and internal hernias [31]. In the present series, excluding cholecystectomy, the rate of reoperation for causes related to the primary surgery is 8.5%, and it has decreased significantly after abandoning the short common channel operation. The number of patients requiring cholecystectomy in the follow-up was 7.7%, comparable to that reported by Higa after gastric bypass [33]; no prophylactic cholecystectomy is recommended.

The incidence of cancer in our study, 4.2% or 6 cases per 1000 patient-years is comparable to that reported by Sjöstrom et al. corresponding to the Swedish Study [34] and close to the reported by Adams et al. in 2009 with more than 6000 patients followed for a mean time of 24 years; they report a total cancer incidence of 3.8% with a total incidence density rate of 3.1 cases per 1000 person-years follow-up [35]. However, in other reported studies, the incidence seems to be much lower, as in the work from Canada of Christou et al., who report a 5-year incidence of 2.03% [36]. In a meta-analysis published in 2014 [37], the cancer incidence density rate was found to be between 1.06 and 1.08 cases per 1000 person-years. In the present series, we have not discarded the neoplastic events presented in the first 3 postoperative years, so the final results could be biased as some cancers were surely present at the moment of surgery. A longer follow-up will surely contribute to the decrease of the total incidence density rate.

Finally, the quality of life after SADI-S is much better than after old biliopancreatic diversions [18, 23], as the stool frequency in the long term is almost normal and only increased after dietary violations.

In summary, we present the long-term evolution of patients submitted to SADI-S, a safe and effective surgery for the treatment of obesity, and its comorbid conditions. This is a prospective study with the limitations of the selection of patients for the procedure and the inclusion of patients submitted to SADI-S with different limb lengths. On the other hand, the strength of the report is the long follow-up of a series of consecutive patients submitted to SADI-S in only one institution, with a 75% follow-up rate at 10 years.
